# Exosomes from endothelial progenitor cells improve outcomes of the lipopolysaccharide-induced acute lung injury

**DOI:** 10.1186/s13054-019-2339-3

**Published:** 2019-02-13

**Authors:** Yue Zhou, Pengfei Li, Andrew J. Goodwin, James A. Cook, Perry V. Halushka, Eugene Chang, Basilia Zingarelli, Hongkuan Fan

**Affiliations:** 1 Department of Pathology and Laboratory Medicine, Medical University of South Carolina, 173 Ashley Ave., MSC 908, CRI Room 610, Charleston, SC 29425 USA; 2 Department of Biopharmaceutics College of Pharmacy, Nanjing University of Chinese Medicine, Nanjing, 210000 China; 3 Division of Pulmonary, Critical Care, Allergy, and Sleep Medicine, Medical University of South Carolina, Charleston, SC 29425 USA; 4 Department of Neurosciences, Medical University of South Carolina, Charleston, SC 29425 USA; 5 Department of Medicine, Medical University of South Carolina, Charleston, SC 29425 USA; 6 Department of Pharmacology, Medical University of South Carolina, Charleston, SC 29425 USA; 7 Department of Obstetrics-Gynecology, Medical University of South Carolina, Charleston, SC 29425 USA; 8 Division of Critical Care Medicine, Cincinnati Children’s Hospital Medical Center, Cincinnati, OH 41073 USA; 9 Department of Regenerative Medicine and Cell Biology, Medical University of South Carolina, Charleston, SC 29425 USA

**Keywords:** Acute lung injury, Exosomes, miR-126, Tight junction protein

## Abstract

**Background:**

The acute respiratory distress syndrome (ARDS) is characterized by disruption of the alveolar-capillary barrier resulting in accumulation of proteinaceous edema and increased inflammatory cells in the alveolar space. We previously found that endothelial progenitor cell (EPC) exosomes prevent endothelial dysfunction and lung injury in sepsis in part due to their encapsulation of miRNA-126. However, the effects of EPC exosomes in acute lung injury (ALI) remain unknown.

**Methods:**

To determine if EPC exosomes would have beneficial effects in ALI, intratracheal administration of lipopolysaccharide (LPS) was used to induce ALI in mice. Lung permeability, inflammation, and the role of miRNA-126 in the alveolar-epithelial barrier function were examined.

**Results:**

The intratracheal administration of EPC exosomes reduced lung injury following LPS-induced ALI at 24 and 48 h. Compared to placebo, intratracheal administration of EPC exosomes significantly reduced the cell number, protein concentration, and cytokines/chemokines in the bronchoalveolar lavage fluid (BALF), indicating a reduction in permeability and inflammation. Further, EPC exosomes reduced myeloperoxidase (MPO) activity, lung injury score, and pulmonary edema, demonstrating protection against lung injury. Murine fibroblast (NIH3T3) exosomes, which do not contain abundant miRNA-126, did not provide these beneficial effects. In human small airway epithelial cells (SAECs), we found that overexpression of miRNA-126-3p can target phosphoinositide-3-kinase regulatory subunit 2 (PIK3R2), while overexpression of miRNA-126-5p inhibits the inflammatory alarmin HMGB1 and permeability factor VEGFα. Interestingly, both miR-126-3p and 5p increase the expression of tight junction proteins suggesting a potential mechanism by which miRNA-126 may mitigate LPS-induced lung injury.

**Conclusions:**

Our data demonstrated that human EPC exosomes are beneficial in LPS-induced ALI mice, in part through the delivery of miRNA-126 into the injured alveolus.

**Electronic supplementary material:**

The online version of this article (10.1186/s13054-019-2339-3) contains supplementary material, which is available to authorized users.

## Background

The acute respiratory distress syndrome (ARDS) is a complex and deadly disease characterized by inflammation and lung permeability leading to alveolar edema, hypoxemia, and organ failure [1, 2]. ARDS occurs in up to 10% of patients admitted to an intensive care unit with mortality rates ranging from 35 to 46% [3, 4]. To date, there are no approved pharmacological treatments for this syndrome. The acute lung injury (ALI) associated with ARDS is characterized by damage and disruption of the epithelial and endothelial layers at the alveolar-capillary barrier and recruitment of inflammatory cells into the alveolar space. The integrity of these layers is, in part, governed by tight junction proteins including claudins and occludins [5] while the activation of endothelial cells and recruitment of inflammatory cells are governed by inflammatory cytokines and chemokines [6]. Thus, therapies which enhance claudin and occludin stability or mitigate local inflammatory cytokine release could reduce the severity of lung injury and improve the patient outcomes.

Endothelial progenitor cells (EPCs) represent a promising therapeutic strategy for ALI [7, 8]. We and other groups have reported that EPCs can mitigate vascular leakage, reduce inflammation, and enhance bacterial clearance in sepsis-induced lung injury, pneumonia, and ALI [9–12]. Recently, exosomes have emerged as an important paracrine mechanism of cell-to-cell communication by facilitating the transfer of miRNAs from one cell to a recipient cell [13]. We have previously shown that EPC exosomes have the ability to transfer miR-126 into endothelial cells resulting in reduced expression of genes relevant to the development of ARDS while fibroblast (NIH3T3) exosomes that do not contain abundant miR-126 exert minimal protective effects [14]. Further, intravenous administration of human EPC exosomes attenuated sepsis-related ALI in mice, an effect dependent upon the presence of miR-126. Exosomes can be stored in a stable manner and, thus, may provide therapeutic advantages over cell-based therapy [15]. However, a complete understanding of the mechanisms of action and therapeutic potential of EPC exosomes in ALI remains an important gap in our knowledge.

We aimed to investigate whether EPC exosomes have biologic impacts on the alveolar epithelium, similar to the endothelium, and whether these effects may also mitigate ALI. We hypothesized that intratracheal administration of EPC exosomes would attenuate ALI through the delivery of miR-126 into epithelial cells leading to the alteration in gene expression. Using a combination of the murine intratracheal lipopolysaccharide (LPS)-induced ALI model, in vitro transfection assays, and next-generation sequencing, we demonstrated that miR-126-containing EPC exosomes decrease the severity of ALI whereas NIH3T3 exosomes with barely detectable levels of miR-126 do not. These findings support a potential therapeutic role for EPC exosomes in ALI through pleiotropic mechanisms.

## Materials and methods

### Isolation and characterization of exosomes

This study was approved by the Institutional Review Board for Human Research at the Medical University of South Carolina. Human EPCs were isolated from the cord blood from a healthy pregnant woman and were cultured as previously described [12]. Briefly, the cord blood samples were collected from the umbilical veins during normal full-term vaginal deliveries, and informed consent was obtained from the mother for all cord blood collections. EPCs were cultured in endothelial basal medium (EBM-2; Lonza, Allendale, NJ, USA) supplemented with EBM-2 SingleQuots (Lonza, Allendale, NJ, USA) containing 10% exosome-depleted fetal bovine serum (FBS; System Biosciences, Palo Alto, CA, USA), 1% penicillin, and streptomycin (GIBCO, Gaithersburg, MD, USA) for 48 h, while NIH3T3 cells were cultured in Dulbecco’s modified Eagle’s medium (DMEM, GIBCO, Gaithersburg, MD, USA) containing 10% exosome-depleted FBS (System Biosciences, Palo Alto, CA, USA), 1% penicillin, and streptomycin (GIBCO, Gaithersburg, MD, USA) for 48 h. Medium was harvested and centrifuged at 2000×*g* for 30 min to remove cells and debris. Exosomes were then isolated from the cell-free medium using the Total Exosomes Isolation Kit following the manufacturer’s instructions (Invitrogen, Asheville, NC, USA) and re-suspended in PBS.

The total protein concentration of the exosomes was measured by detergent-compatible (DC) protein assay (Bio-Rad, Hercules, CA, USA). The size distribution and the total number of exosomes were analyzed by nanoparticle tracking analysis (NTA) with ZetaView PMX 120 (Particle Metrix, Meerbusch, Germany). Exosome markers, such as tetraspanin proteins CD9, CD63, and CD81, were determined by western blot. Each experiment was carried out in triplicate.

### Lipopolysaccharide-induced acute lung injury model

Previously, we reported that EPC exosomes exert protective effects in a cecal ligation and puncture model which is a clinically relevant murine model of sepsis. To further explore the effects of EPC exosomes in a murine ALI model, intratracheal instillation of LPS was used to induce ALI. Investigations conformed to the Guide for the Care and Use of Laboratory Animals published by the NIH and were approved by the Institutional Animal Care and Use Committee at the Medical University of South Carolina. CD-1 outbred mice (aged 7–8 weeks) were housed in a pathogen-free environment. The mice underwent intratracheal instillation of either 25 μg LPS diluted in 75 μl PBS as described previously [16] or 75 μl PBS. Four hours after acute lung injury induction, the mice were treated with 70 μg of EPC exosomes or negative control NIH3T3 exosomes or PBS separately through intratracheal administration. Thus, four experimental groups were created: (1) PBS control, (2) LPS+PBS, (3) LPS+EPC-exo, and (4) LPS+3T3-exo. Subsequent experiments examined three to seven mice per group. Bronchoalveolar lavage fluid (BALF) and perfused lung tissues for myeloperoxidase (MPO) activity and Evans blue assay were collected at 24 h as described below, and formalin-fixed paraffin-embedded histological lung tissues were collected at 48 h after lung injury. All the samples were stored at − 80 °C or 4 °C until analysis.

### Assessment of lung inflammation

Lung inflammation was compared between experimental groups using the following methods: (1) measurement of BALF cell count, (2) measurement of BALF cytokines and chemokines, and (3) measurement of lung tissue myeloperoxidase activity. BALF was collected from mice in each group 24 h after LPS instillation. After euthanasia, the thorax was opened to expose the trachea. The trachea was cannulated with a 20-g angiocatheter and lavaged four times with cold PBS (0.75 ml) using a 1-ml syringe. The BALF was centrifuged at 600×*g* for 5 min to pellet cells. The cell pellet was re-suspended in 500 μl red blood cell lysis buffer and centrifuged at 600×*g* for 5 min. Cell pellets were re-suspended in 500 μl PBS, and immune cells were quantified using a Countess II Automated Cell Counter (Thermo Fisher Scientific, Waltham, MA, USA). The supernatant was collected and analyzed for cytokine and chemokine levels using the pro-inflammatory focused 32-plex (Eve Technologies, Calgary, AB). The remaining supernatant was aliquoted and frozen at − 80 °C for additional experiments.

Myeloperoxidase activity was determined in lung tissue as an index of neutrophil accumulation as previously described [17, 18]. Briefly, lung tissues were perfused, weighed, and homogenized in 1 ml potassium phosphate buffer (50 Mm, pH 6.0). The homogenized tissues were centrifuged for 15 min at 10000 rpm, and the supernatant was discarded. The tissues were re-suspended in 1 ml potassium phosphate solution (50 mM) containing 0.5% hexadecyl-trimethylammonium and sonicated for 20 s. The samples were frozen and thawed twice and centrifuged for 10 min at 10000 rpm. The supernatants (10 μl) were combined with 80 μl 0.75 mM H_2_O_2_ (Sigma, St. Louis, MO, USA) and 110 μl TMB solution (2.9 mM TMB in 14.5% DMSO and 150 mM sodium phosphate buffer at pH 5.4), and the plate was incubated at 37 °C for 5 min. The reaction was stopped by adding 50 μl H_2_SO_4_ (2 M, Sigma, St. Louis, MO, USA), and the absorption was measured at 450 nm. The quantification of MPO was calculated from a MPO standard curve and was expressed in units per gram of the tissue.

### Measurement of lung vascular leak

Lung vascular leak was measured in each experimental group using (1) Evans blue assay in lung tissue, (2) lung water content, and (3) BALF protein concentrations. The Evans blue dye assay was performed as described previously [19]. Briefly, the mice were administered 1% Evans blue dye solution (Sigma, St. Louis, MO, USA) in saline via tail vein injection. After 40 min, the mice were sacrificed and perfused via the heart, and the lung tissues were collected. The lung weights were measured and placed in 1 ml of formamide (Avantor, Center Valley, PA, USA) at 60 °C for 24 h to extract Evans blue dye. The samples were centrifuged at 2000 rpm for 10 min, and the supernatants were collected. The concentrations of Evans blue dye in the supernatants were quantified by measuring absorbance at 620 nm and calculated from a standard curve by a plate reader.

For lung water content, the left lung was harvested and weighed to measure a wet weight in each group. The wet lung was then dried in an oven at 60 °C for 48 h and re-weighed as dry weight. The lung water content was calculated as the ratio of wet weight to dry weight. Protein levels in the BALF supernatant were determined by DC protein assay (Bio-Rad, Hercules, CA, USA).

### Lung histology and lung injury score

The lung tissues were collected from mice at 48 h after LPS instillation. The lungs were inflated with 10% buffered formalin, fixed with 10% buffered formalin, embedded in paraffin, and cut into 5-μm sections. Tissue sections were stained with hematoxylin and eosin (H&E), evaluated, and scored by a pathologist who was blinded to the experimental groups. To evaluate the lung jury, seven independent random lung fields were evaluated per mouse for neutrophils in alveolar spaces, neutrophils in the interstitial spaces, hyaline membranes, proteinaceous debris filling the airspaces, and alveolar septal thickening and weighted according to the relevance ascribed by the official American Thoracic Society workshop report on features and measurements of experimental acute lung injury in animals [20]. The resulting injury score is a continuous value between 0 and 1.

### Human small airway epithelial cell culture and miR-126 transfection

To determine the potential effects of exosomal miR-126 on epithelial gene expression, small airway epithelial cells (SAECs) were transfected with synthetic miR-126. SAECs were cultured in human collagen type IV-coated flasks (Sigma, St. Louis, MO) supplemented with SAGM Bullet kit culture medium (Lonza, Allendale, NJ, USA). Cells were seeded into 12-well plates and then transfected with a miR-126-3p mimic (40 nM), miR-126-5p mimic (40 nM), or control miRNA (40 nM) for 48 h using human airway epithelial cell avalanche transfection reagent according to the manufacturer’s instructions.

At 24 h after transfection, the cells were then stimulated with LPS (100 ng/ml, Sigma, St. Louis, MO, USA) for 24 h. The total RNA was extracted from cells, which was used to do RNA sequencing analysis as well as measurement of mRNA levels by real-time polymerase chain reaction (RT-PCR) described below.

### RNA sequencing and pathway analysis

To determine the effect of miR-126-3p and miR-126-5p on gene expression profile in SAECs, we transfected SAECs with miR-126-3p and miR-126-5p or control siRNAs for 48 h and total RNAs were isolated using RNeasy plus kit (QIAGEN, Germantown, MD, USA) following the manufacturer’s instructions. Extracted RNA was used to prepare next-generation sequencing (NGS) libraries, and the sequencing was performed on an Illumina HiSeq 2500 instrument at the MUSC Genomic Sequencing Core Facility or by Novogene (Chula Vista, CA, USA). The pathway analysis was performed with the Genomic Sequencing Core Facility.

### Real-time PCR

Total RNA extracted from SAECs was also used to perform RT-PCR to validate differentially expressed mRNA identified in the RNAseq analysis. The RNA (10 μl per reaction) was reverse transcribed using the High Capacity cDNA Reverse Transcription Kits (Thermo Fisher Scientific, Waltham, MA, USA). Following cDNA synthesis, the levels of mRNA were determined by CFX96 Real-Time PCR system (Bio-Rad, Hercules, CA, USA) using SYBR green qPCR master mix (QIAGEN, Germantown, MD, USA) according to the manufacturer’s instructions. Data were analyzed with 2^−ΔΔCt^ value calculation, using GAPDH for normalization.

### Western blot

EPC exosomes and NIH3T3 exosomes were lysed with ice-cold radioimmunoprecipitation assay (RIPA) lysis buffer (Abcam, Cambridge, MA, USA) containing protease and phosphatase inhibitors (Cell Signaling, Boston, MA, USA). All lysed samples were kept on ice for 30 min and centrifuged for 10 min at 4 °C at 10,000×*g*. The cell lysates were collected, and protein concentrations were measured using a DC protein assay (Bio-Rad, Hercules, CA, USA). Approximately 20 μg of exosomes protein was loaded into each lane for western blot. All exosome-specific primary antibodies including anti-CD9, anti-CD63, and anti-CD81 (System Biosciences, Palo Alto, CA, USA) were used at 1:1000 dilution, and exosome-validated peroxidase-labeled secondary antibody was at 1: 20000 dilution. The immunoreactive protein bands were visualized by ECL detection kit (GE Healthcare, Pittsburgh, PA, USA) and analyzed using ImageJ software.

### Statistical analysis

The in vitro experiments were performed at least three independent times. The data were analyzed using GraphPad Prism 7.01 software and represented as mean ± SE. Means of multiple groups were compared by one-way analysis of variance (ANOVA). Independent sample *t* test was performed to compare the means between two different groups. A value of *p* < 0.05 was considered statistically significant.

## Results

### Characterization of exosomes

**Fig. 1:**
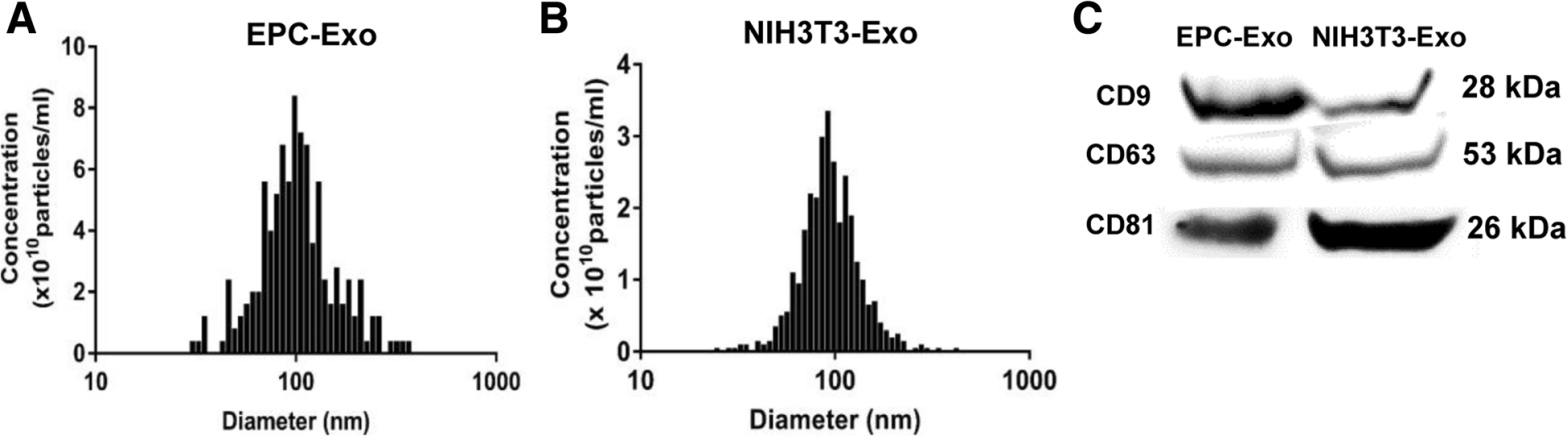
Characterization of exosomes derived from human EPCs and NIH3T3 fibroblasts. **a**, **b** Exosome size distribution and concentration were measured by nanoparticle tracking analysis (NTA) with ZetaView. **c** Detection of exosome markers including CD9, CD63, and CD81 in EPC exosomes and NIH3T3 exosomes by western blot

Nanoparticle tracking analysis (NTA) showed that the concentration of isolated EPC exosomes is about 1.7 × 10^11^ particles/ml and NIH3T3 exosomes are about 4.7 × 10^10^ particles/ml. Both types of exosomes showed a similar size distribution profile with 97% of isolated particles within the 30–120 nm range (Fig. 1a, b). Western blot further confirmed that tetraspanin proteins (CD9, CD63, CD81) were present in all samples (Fig. 1c). These characterization data are consistent with successful exosome isolation as previously described [21].

### EPC exosomes decreased cell counts, protein concentration, and cytokines/chemokines of BALF in LPS-induced acute lung injury

**Fig. 2:**
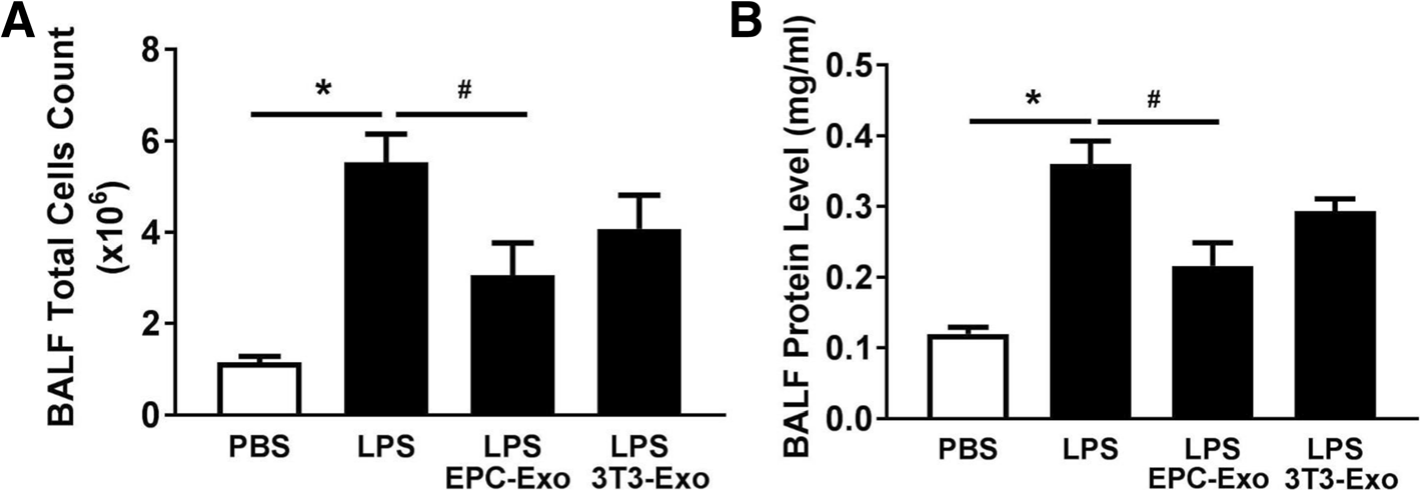
Therapeutic effects of EPC exosomes on bronchoalveolar lavage fluid (BALF) cell counts and protein concentration in LPS-induced acute lung injury. Mice were subjected to acute lung injury (ALI) by LPS instillation and treated with either EPC exosomes or NIH3T3 exosomes or PBS at 4 h after injury. BALF total cell counts (**a**) and BALF protein concentration (**b**) were determined at 24 h after injury. *p < 0.05 compared with the PBS group; ^#^*p* < 0.05 compared with the LPS group. *n* = 6–7 mice per group. Results are represented as mean ± SE

Increased BALF cell number and protein concentration are representative of augmented endothelial and epithelial permeability. CD-1 mice underwent intratracheal instillation of LPS and were treated intratracheally with EPC exosomes or NIH3T3 exosomes at 4 h post-injury. The cell counts and protein concentration levels in the BALF were determined. LPS installation significantly increased the total cell counts and protein concentration levels in BALF compared with PBS instillation. These effects were mitigated by intratracheal administration of EPC exosomes (*p* < 0.05) but not by administration of NIH3T3 exosomes (Fig. 2a, b).

**Fig. 3:**
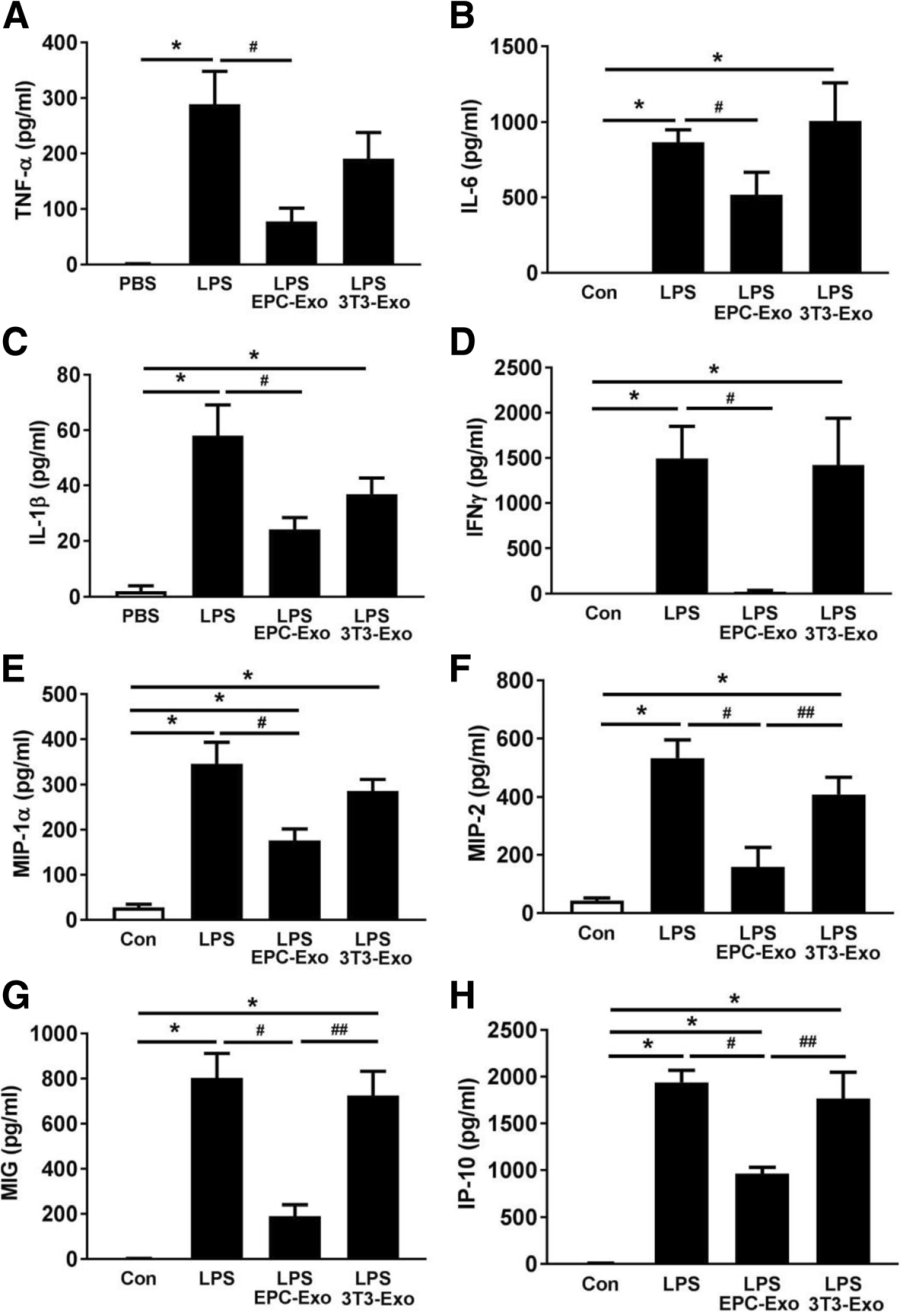
Therapeutic effects of EPC exosomes on BALF cytokines and chemokines in LPS-induced acute lung injury. BALF cytokines TNF-α (**a**), IL-6 (**b**), IL-1β (**c**), and IFNγ (**d**) and chemokines MIP-1 (**e**), MIP-2 (**f**), MIG (**g**), and IP-10 (**h**) were determined by mouse cytokine and chemokine array at 24 h after LPS installation. *p < 0.05 compared with the PBS group; ^#^*p* < 0.05 compared with the LPS group. ^##^*p* < 0.05 compared with the LPS+EPC exosomes group. *n* = 4 mice per group. Results are represented as mean ± SE

Moreover, LPS also induced lung inflammation as evidenced by increased cytokines and chemokines including tumor necrosis factor (TNF)-α, interleukin (IL)-6, IL-1β, interferon (IFN)γ, macrophage inflammatory proteins (MIP)-1, MIP2, monokine induced by gamma interferon (MIG), and interferon gamma-induced protein (IP)-10 in the BALF compared to PBS group. EPC exosome treatment significantly attenuated these increases of inflammatory mediators (*p* < 0.05; Fig. 3a–h), while administration of negative control NIH3T3 exosomes at the same dose had no beneficial effects in LPS-induced cytokine and chemokine production (*p* > 0.05; Fig. 3a–h). These data suggest that EPC exosome treatment reduced the damage to the alveolar-capillary barrier and cytokine and chemokine release caused by LPS.

### EPC exosomes reduced alveolar edema**,** lung injury, and lung neutrophil infiltration in ALI mice

**Fig. 4:**
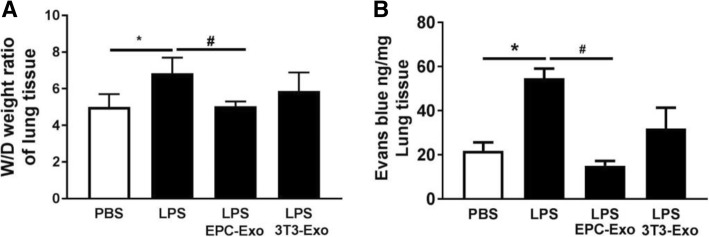
Therapeutic effects of EPC exosomes on alveolar edema in LPS-induced acute lung injury. Lung water content was calculated as the ratio of wet weight to dry weight (**a**), and vascular leakage in lung tissue was measured via injecting Evans blue dye at 24 h after LPS instillation (**b**). *p < 0.05 compared with the PBS group; ^#^*p* < 0.05 compared with the LPS group. *n* = 4–6 mice per group. Results are represented as mean ± SE

**Fig. 5:**
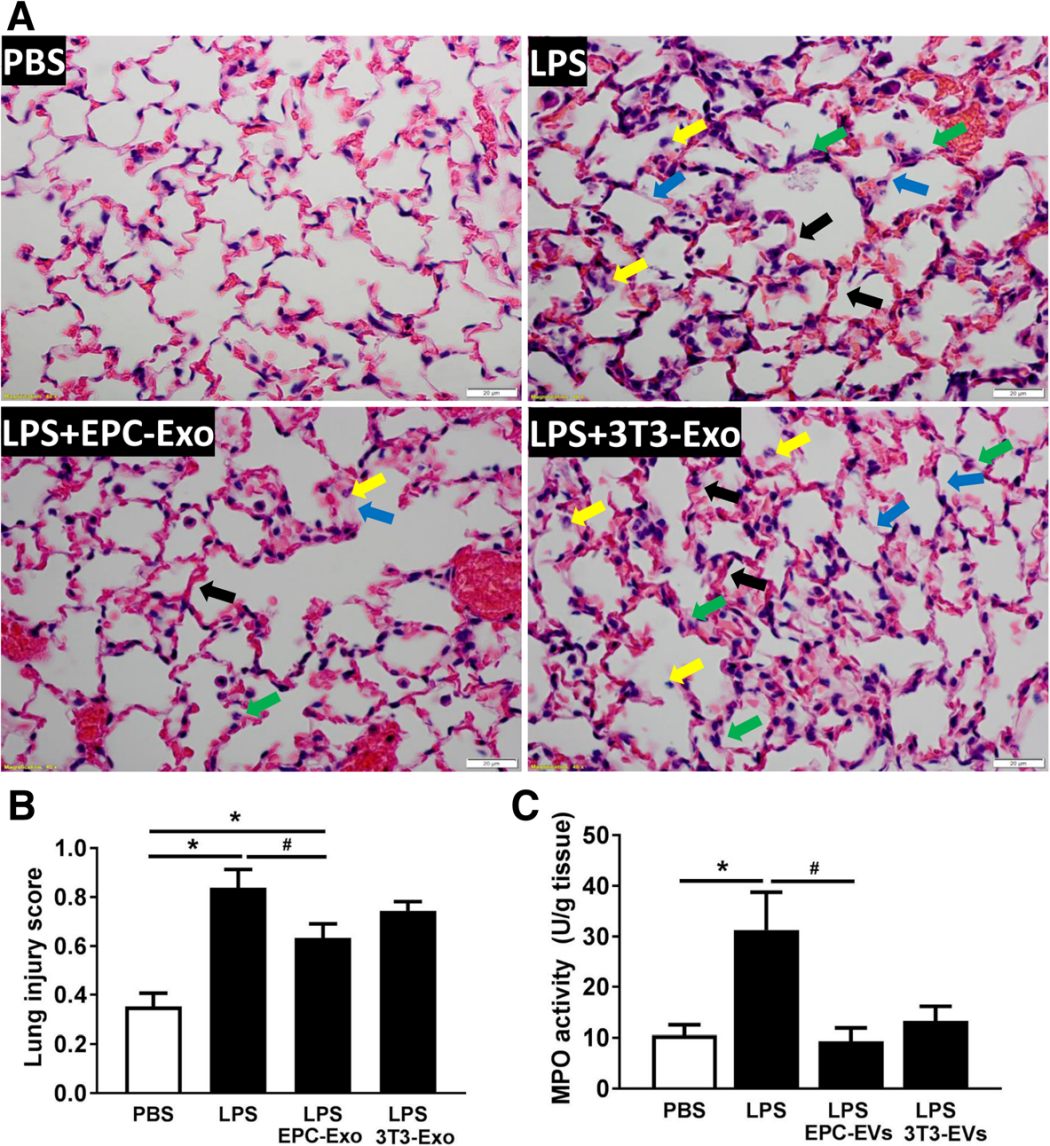
Therapeutic effects of EPC exosomes on LPS-induced acute lung injury by histological analysis and myeloperoxidase (MPO) activity. Lung (**a**) sections were stained with H&E and examined histologically at 48 h after LPS instillation. The representative sections are shown at × 400 original magnification, and scale bars are 20 mm. The PBS group showed normal lung tissue including thin alveolar walls and few alveolar macrophages. Yellow arrows indicate neutrophils in the alveolar space, green arrows indicate neutrophils in the interstitial space, blue arrows indicates hyaline membranes, and black arrows indicate thickening of the alveolar walls. Lung injury scores (**b**) were assessed. *p < 0.05 compared with the PBS group; ^#^*p* < 0.05 compared with the LPS group. *n* = 4 mice per group. Results are represented as mean ± SE. MPO activity (**c**) in the lung tissue were measured at 24 h after LPS instillation. *p < 0.05 compared with the PBS group; ^#^*p* < 0.05 compared with the LPS group. *n* = 3–6 mice per group. Results are represented as mean ± SE

We subsequently investigated the effect of EPC exosomes on epithelial barrier integrity and alveolar edema. Lung water content expressed as wet/dry weight was significantly increased in ALI mice, which was decreased by EPC exosome treatment (*p* < 0.05, Fig. 4a). Evans blue dye was used to determine the changes in alveolar permeability. Mice with ALI exhibited a marked increase in alveolar edema assessed by Evans blue tissue dispersion, which was reversed by EPC exosome treatment (*p* < 0.05; Fig. 4b). Histologic examination of the PBS group revealed normal mouse lung characterized by thin alveolar walls with occasional alveolar macrophages and rare neutrophils. However, the mice treated with LPS demonstrated significantly increased neutrophils in both the alveolar and interstitial spaces, hyaline membrane formation, and thickening of the alveolar walls (Fig. 5a). These observations were significantly reduced after treatment with EPC exosomes, whereas treatment with NIH3T3 exosomes had no effect. These observations were confirmed by lung injury score evaluation (*p* < 0.05; Fig. 5b) and suggested that intratracheal administration of EPC exosomes protects against lung injury while NIH3T3 exosomes do not.

We also examined MPO activity to evaluate neutrophil accumulation in the lung tissue. LPS administration significantly increased the MPO activity, whereas MPO activity was significantly reduced by EPC exosome treatment (*p* < 0.05; Fig. 5c).

### MiR-126-3p and miR-126-5p augmented tight junction protein levels in lung SAECs

**Fig. 6:**
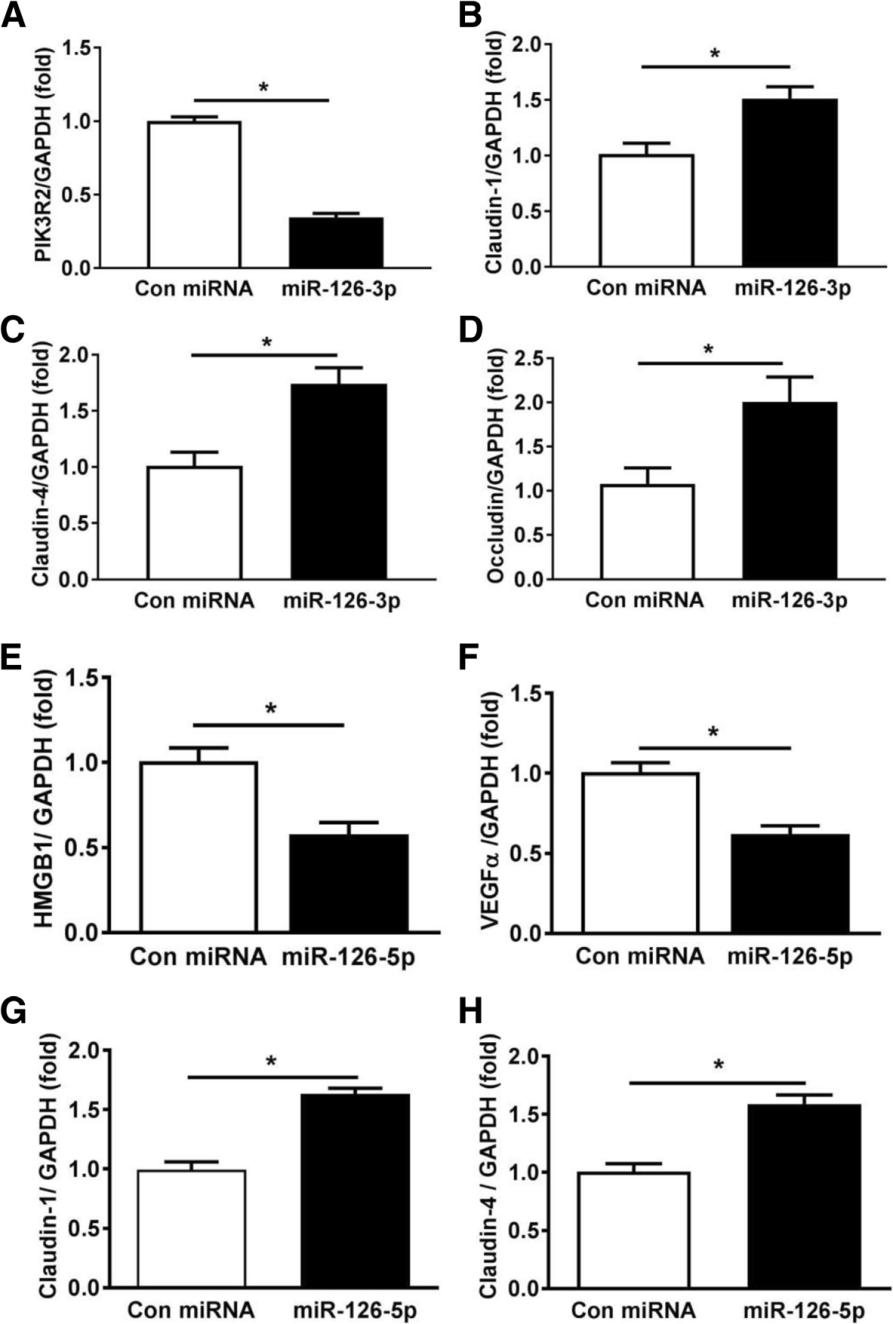
miRNA-126-3p and miRNA-126-5p target PIK3R2, HMGB1, and VEGFα and regulate tight junction protein expression levels in human small airway epithelial cells (SAECs). To verify RNA sequencing data (Tables 1 and 2), SAECs were transfected with either miR-126-3p mimic, miR-126-5p mimic, or control miRNA for 48 h. PIK3R2, the target of miR-126-3p (**a**), epithelial tight junction claudin1 (**b**), claudin4 (**c**), and occludin (**d**) mRNA levels were measured by RT-qPCR. HMGB1 (**e**) and VEGFα (**f**), the targets of miR-126-5p; epithelial tight junction claudin1 (**g**); and claudin4 (**h**) mRNA levels were determined by RT-qPCR. GAPDH served as an internal control. *p < 0.05 compared with the control group; the experiments were performed at least three independent times. Results are represented as mean ± SE

As miR-126-3p and miR-126-5p are highly abundant in EPC exosomes but not in NIH3T3 exosomes [14], we examined how miR-126-3p and miR-126-5p regulate lung alveolar epithelial gene expression by RNA sequencing. SAECs were transfected with either control miRNA, miR-126-3p mimic, or miR-126-5p mimic for 48 h, and total RNA was isolated for RNA sequencing analysis. The RNA sequencing results showed more than 4500 genes were significantly altered by overexpression of miR-126-3p (Additional file 1) and more than 6000 genes regulated by miR-126-5p (Additional file 2) in SAECs. Pathway analysis showed that cell adhesion molecules were significantly regulated by both miR-126-3p and miR-126-5p. MiR-126-3p transfection significantly decreased PIK3R2 mRNA levels, which is a known target of miR-126-3p [22] (Table 1). MiR-126-5p similarly significantly decreased its targets high-mobility group protein (HMGB)1 and vascular endothelial growth factor (VEGF)α (Table 2). These results suggested that SAECs were successfully transfected with miR-126-3p or miR-126-5p. Moreover, the sequencing data showed that mRNA expression levels of several tight junction genes including claudin1 and claudin4 were significantly increased by both miRNA-126-3p and miRNA-126-5p (Tables 1 and 2), while occludin levels were increased by miR-126-3p (Table 1). Further, these data were validated by the RT-qPCR (*p* < 0.05; Fig. 6a–h).

**Table 1:** SAEC genes regulated by miR-126-3p with relevance to the lung barrier integrity by RNA sequencing analysis

Category	Gene	Fold change	*p* value
Epithelial tight junction	Claudin1	1.5 ± 0.06	2.49 × 10^−32^
	Claudin4	3.1 ± 0.19	5.93 × 10^−5^
	Occludin	3.6 ± 0.13	1.45 × 10^−14^
Epithelial activation/integrity	PIK3R2	0.43 ± 0.03	3.82 × 10^−41^

Only selected relevant genes were listed here; the other genes altered by miR-126-3p were list in Additional file 1

**Table 2:** SAEC genes regulated by miR-126-5p with relevance to the lung barrier integrity and inflammation by RNA sequencing analysis

Category	Gene	Fold change	*p* value
Epithelial tight junction	Claudin1	1.8 ± 0.03	1.65 × 10^−11^
	Claudin4	2.2 ± 0.12	1.56 × 10^−10^
Vascular permeability factor	VEGFα	0.28 ± 0.01	8.34 × 10^−28^
Inflammatory alarmin	HMGB1	0.66 ± 0.02	2.01 × 10^−7^

Only selected relevant genes were listed here; the other genes altered by miR-126-5p were list in Additional file 2

### MiRNA-126-3p and miRNA-126-5p maintained the lung alveolar epithelial barrier integrity

**Fig. 7:**
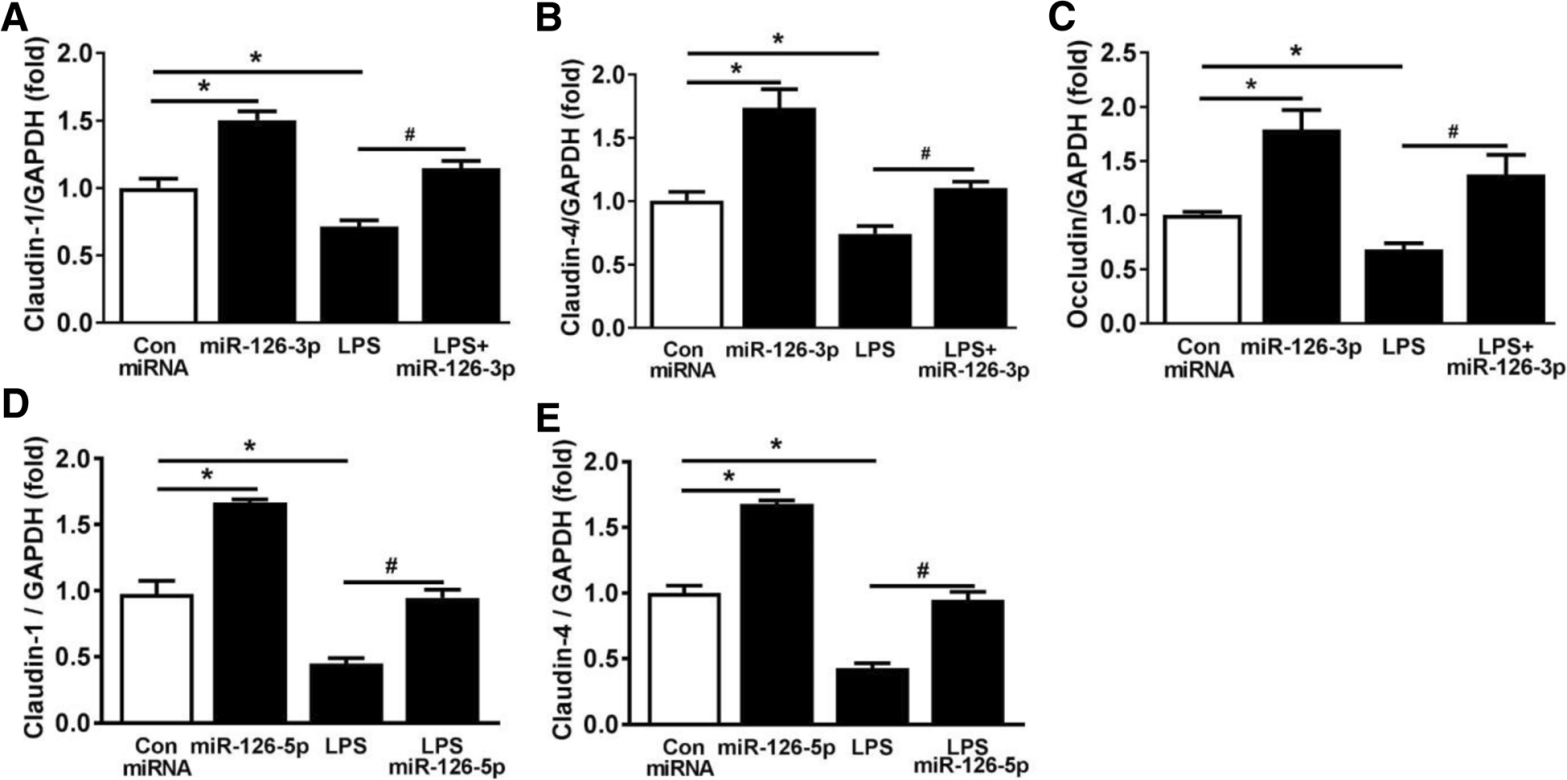
miRNA-126-3p and miRNA-126-5p regulate the expression levels of cell tight junction proteins in LPS-stimulated SAECs. SAECs were transfected with either miR-126-3p mimic, miR-126-5p mimic, or control miRNA for 48 h and stimulated with LPS (100 ng/ml) for 24 h. mRNA levels of claudin1 (**a**, **d**), claudin4 (**b**, **e**), and occludin (**c**) in SAECs were measured by RT-qPCR. GAPDH served as an internal control. *p < 0.05 compared with the control group; ^#^*p* < 0.05 compared with the LPS group. The experiments were performed at least three independent times. Results are represented as mean ± SE

The tight junction proteins claudin1, claudin4, and occludin expression levels affect the alveolar epithelial barrier function [23, 24]. We examined the potential role of miR-126-3p and miR-126-5p in regulating epithelial barrier integrity under normal conditions and with LPS stimulation. We observed that LPS markedly decreased the mRNA expression levels of claudin1, claudin4, and occludin in SAECs (*p* < 0.05; Fig. 7a–e), while overexpression with either miR-126-3p or miR-126-5p attenuated these changes (*p* < 0.05; Fig. 7a–e). These data demonstrate that miR-126-3p and miR-126-5p may prevent the loss of epithelial tight junctions associated with ALI suggesting a possible mechanism by which EPC exosomes mitigate alveolar edema and lung injury.

## Discussion

EPC exosomes and extracellular vesicles exhibit a number of beneficial effects in endothelial health in vitro and in animal models of sepsis, acute kidney injury, and ischemia [14, 25, 26]. Further, our previous work demonstrated that EPC exosome treatment can target HMGB1 and VCAM1 expression, thereby reducing lung microvascular endothelial inflammation and dysfunction through the delivery of miRNA-126 [14]. In this study, we examined the impact of EPC exosomes on ALI-induced alveolar epithelial damage. EPC exosome intratracheal administration attenuated lung injury through reduction of local inflammatory cytokines, pulmonary permeability, and neutrophil migration. Subsequently, we demonstrated that miR-126-3p and miR-126-5p, which are abundant in EPC exosomes, increase epithelial tight junction protein expression while decreasing target genes with relevance to ALI such as phosphoinositide-3-kinase regulatory subunit 2 (PIK3R2), HMGB1, and VEGFα.

Acute lung injury is a complex derangement of pulmonary physiology involving a number of cell types including the endothelium, epithelium, and inflammatory cells such as neutrophils. Endothelial activation and dysfunction contribute to increased capillary permeability and alveolar edema, and our previous work demonstrated that EPC exosomes can mitigate this response through the transfer of miR-126 [14]. Here, we have demonstrated that EPC exosomes delivered intratracheally also mitigate lung injury and that these exosomes can enter epithelial cells and modulate the expression of a number of relevant genes including cytokines, VEGFα, and tight junction components. These findings suggest that EPC exosomes can reduce not only endothelial but also epithelial dysfunction in ALI. Further, in the setting of improved alveolar-capillary barrier function and reduced inflammatory cytokines, neutrophil migration was also reduced in the EPC exosome-treated mice. Taken together, these data significantly enhance the potential of EPC exosomes as a therapeutic in ALI as they can impact several relevant cell types through multiple delivery approaches.

We previously found that human EPC exosomes contain highly abundant levels of miRNA-126, like their parent cell, and that miRNA-126 mediates the restorative effect of EPCs on endothelial dysfunction [27]. Although miR-126 expression is strongly associated with endothelial cells, we hypothesized that it may also play a significant role in epithelial homeostasis. This theory is supported by our observations that miR-126-depleted exosomes from NIH3T3 cells do not improve lung injury from LPS and that miR-126-3p and miR-126-5p increase tight junction protein expression while reducing the expression of genes related to permeability. The NIH3T3 exosomes do show some beneficial effect in some measurement such as IL-1β levels and MPO activity. It is possible that other exosomal components beside miR-126 may exert protective effects as well, which merit further investigation.

These findings are similar to what others have previously reported. Overexpression of miRNA-126 decreased lung endothelium injury and mortality in endotoxin-induced mice [28], and miR-126 has been shown to reduce EC barrier disruption by regulating tight junction protein expression including zonula occluden-1 (ZO-1), occludin, and claudin [29, 30]. Moreover, a recent study reported that miRNA-126-3p preserved endothelial barrier integrity via targeting PIK3R2 with the activation of the Akt pathway [22, 31]. Of note, activation of the Akt signaling pathway can further increase the tight junction proteins such as claudin and occludin [23, 32]. Finally, HMGB1 has been shown to impair epithelial barrier function via inhibition of cell junction proteins including caludin1 [33]. Our study is the first to our knowledge to identify that miR-126 delivered by EPC exosomes exerts similar effects in the epithelium during ALI. Thus, EPC exosomes may exert their therapeutic potential through the restoration of alveolar barrier integrity by inhibiting PIK3R2 and HMGB1 and increasing the levels of tight junction proteins including claudin1, claudin4, and occludin. Additionally, exosome-mediated delivery of miRNA-126-5p inhibits VEGFα expression further attenuating ALI-induced permeability. Therefore, the delivery of miR-126 through EPC exosomes could serve as the basis of a novel therapeutic in ARDS through pleiotropic effects on gene expression in a number of cell types.

This study has limitations. (1) We used commercial kits to isolate exosomes, and the yield may contain protein-bound miRNA contamination. However, we used the same method to isolate the control NIH3T3 exosomes in order to mitigate any impact on differences between the isolated exosomes. (2) EPC exosomes were administered 4 h after LPS instillation in a relatively early stage of lung injury. Although we did not examine the efficacy of treating with EPC exosomes at later time points here, our previous work has demonstrated that EPCs can mitigate organ injury and death when administered up to 24 h after the onset of experimental sepsis. These data and an increasing focus on early and preventative treatment strategies for ARDS (http://petalnet.org/) suggest that EPC exosomes could have therapeutic potential in human ARDS. (3) Human small airway epithelial cells may not accurately reflect the gene expression patterns of alveolar epithelial cells. SAECs are commonly used as surrogates for alveolar epithelial cells due to the challenges of isolating and maintaining the alveolar cells. While these data provide proof of concept information that miR-126 can augment epithelial tight junction protein expression, they need to be further confirmed in alveolar epithelial cells. (4) We first discovered that miR-126 can upregulate the tight junction proteins and downregulate the expression levels of PIK3R2, HMGB1, and VEGFα in normal and/or LPS-stimulated SAECs. Since microRNAs could regulate different targets, the off-target effects of miR-126 remain to be further investigated. For instance, the sequencing results revealed several differentially expressed genes such as serpin peptidase inhibitor (SERPINB4), thymidine kinase 1 (TK1), CXCL14, and Krüppel-like factor 4 (KLF4) of unclear significance in ARDS but which could represent meaningful off-target effects. The impact of these effects merits further investigation. Also, further mechanistic studies to maximize the therapeutic effect of EPC exosomes on the pulmonary epithelium is still needed. In this process, a novel and safe therapy for ALI/ARDS might eventually emerge.

These data have translational value as they demonstrate that intratracheal delivery of EPC exosomes can mitigate lung injury potentially through the delivery of miR-126 to epithelial cells. They complement our previously published work demonstrating that intravenous EPC exosomes attenuate sepsis-related ALI [14] while also suggesting that aerosolization could be a potential alternative route of delivery. Further investigation into the pharmacokinetics, toxicity, off-targeting effects, and efficacy in non-LPS-induced ALI will be important future steps before such a therapy could be effectively translated.

## Conclusions

We demonstrated that intratracheal administration of EPC exosomes exerted a beneficial effect in LPS-induced ALI versus exosomes derived from fibroblasts. Further, we identified that the transfer of miRNA-126 may be a novel mechanism by which these exosomes can restore injured alveolar epithelium.

## Additional files

Additional file 1: The RNA sequencing results showed genes were significantly altered by overexpression of miR-126-3p in SAECs. Human small airway epithelial cells (SAECs) were transfected with either control miRNA or miR-126-3p mimic for 48 h, and total RNA was isolated for RNA sequencing analysis. Genes significantly altered by miR-126-3p were listed. (XLSX 366 kb)

Additional file 2: The RNA sequencing results showed genes were significantly altered by overexpression of miR-126-5p in SAECs. Human small airway epithelial cells (SAECs) were transfected with either control miRNA or miR-126-5p mimic for 48 h, and total RNA was isolated for RNA sequencing analysis. Genes significantly altered by miR-126-5p were listed. (XLSX 433 kb)

## Abbreviations

ALI: Acute lung injury
ARDS: Acute respiratory distress syndrome
BALF: Bronchoalveolar lavage fluid
DMEM: Dulbecco’s modified Eagle’s medium
EPC: Endothelial progenitor cell
H&E: Hematoxylin and eosin
HMGB: High-mobility group protein
IFN: Interferon
IL: Interleukin
IP: Interferon gamma-induced protein
LPS: Lipopolysaccharide
MIG: Monokine induced by gamma interferon
MIP: Macrophage inflammatory protein
MPO: Myeloperoxidase
NGS: Next-generation sequencing
NTA: Nanoparticle tracking analysis
PIK3R2: Phosphoinositide-3-kinase regulatory subunit 2
RT-PCR: Real-time polymerase chain reaction
SAECs: Human small airway epithelial cells
TNF: Tumor necrosis factor
VEGF: Vascular endothelial growth factor

## References

[1] Thompson BT, Chambers RC, Liu KD (2017). Acute respiratory distress syndrome. N Engl J Med.

[2] Rawal G, Yadav S, Kumar R (2018). Acute respiratory distress syndrome: an update and review. J Transl Int Med.

[3] Pais FM, Sinha P, Liu KD, Matthay MA (2018). Influence of clinical factors and exclusion criteria on mortality in ARDS observational studies and randomized controlled trials. Respir Care.

[4] Bellani G, Laffey JG, Pham T, Fan E, Brochard L, Esteban A, Gattinoni L, van Haren F, Larsson A, McAuley DF, Ranieri M, Rubenfeld G, Thompson BT, Wrigge H, Slutsky AS, Pesenti A, Investigators LS, Group ET. Epidemiology (2016). Patterns of care, and mortality for patients with acute respiratory distress syndrome in intensive care units in 50 countries. JAMA.

[5] Gunzel D, Yu AS (2013). Claudins and the modulation of tight junction permeability. Physiol Rev.

[6] Chollet-Martin S, Jourdain B, Gibert C, Elbim C, Chastre J, Gougerot-Pocidalo MA (1996). Interactions between neutrophils and cytokines in blood and alveolar spaces during ARDS. Am J Respir Crit Care Med.

[7] Lee JW, Zhu Y, Matthay MA (2012). Cell-based therapy for acute lung injury: are we there yet?. Anesthesiology.

[8] Kawasaki T, Nishiwaki T, Sekine A, Nishimura R, Suda R, Urushibara T, Suzuki T, Takayanagi S, Terada J, Sakao S, Tatsumi K (2015). Vascular repair by tissue-resident endothelial progenitor cells in endotoxin-induced lung injury. Am J Respir Cell Mol Biol.

[9] Cao JP, He XY, Xu HT, Zou Z, Shi XY (2012). Autologous transplantation of peripheral blood-derived circulating endothelial progenitor cells attenuates endotoxin-induced acute lung injury in rabbits by direct endothelial repair and indirect immunomodulation. Anesthesiology.

[10] Lam CF, Liu YC, Hsu JK, Yeh PA, Su TY, Huang CC, Lin MW, Wu PC, Chang PJ, Tsai YC (2008). Autologous transplantation of endothelial progenitor cells attenuates acute lung injury in rabbits. Anesthesiology.

[11] Mao M, Wang SN, Lv XJ, Wang Y, Xu JC (2010). Intravenous delivery of bone marrow-derived endothelial progenitor cells improves survival and attenuates lipopolysaccharide-induced lung injury in rats. Shock.

[12] Fan H, Goodwin AJ, Chang E, Zingarelli B, Borg K, Guan S, Halushka PV, Cook JA. Endothelial progenitor cells and a SDF-1alpha analogue synergistically improve survival in sepsis. Am J Respir Crit Care Med. 2014;15:189(12):1509-19. 10.1164/rccm.201312-2163OC PMC4226015 24707934

[13] Valadi H, Ekstrom K, Bossios A, Sjostrand M, Lee JJ, Lotvall JO (2007). Exosome-mediated transfer of mRNAs and microRNAs is a novel mechanism of genetic exchange between cells. Nat Cell Biol.

[14] Zhou Y, Li P, Goodwin AJ, Cook JA, Halushka PV, Chang E, Fan H (2018). Exosomes from endothelial progenitor cells improve the outcome of a murine model of sepsis. Mol Ther.

[15] Conlan RS, Pisano S, Oliveira MI, Ferrari M, Mendes Pinto I (2017). Exosomes as reconfigurable therapeutic systems. Trends Mol Med.

[16] Guo C, Goodwin AJ, Buie JN, Cook JA, Halushka PV, Argraves K, Zingarelli B, Zhang XK, Wang L, Fan H. A stromal cell-derived factor 1 alpha analogue improves endothelial cell function in lipopolysaccharide-induced acute respiratory distress syndrome. Mol Med. 2016;22:115-23. 10.2119/molmed.2015.00240 PMC5004713 27031787

[17] Pulli B, Ali M, Forghani R, Schob S, Hsieh KL, Wojtkiewicz G, Linnoila JJ, Chen JW (2013). Measuring myeloperoxidase activity in biological samples. PLoS One.

[18] Fan H, Zingarelli B, Peck OM, Teti G, Tempel GE, Halushka PV, Spicher K, Boulay G, Birnbaumer L, Cook JA (2005). Lipopolysaccharide- and gram-positive bacteria-induced cellular inflammatory responses: role of heterotrimeric Galpha(i) proteins. Am J Physiol Cell Physiol.

[19] Radu M, Chernoff J. An in vivo assay to test blood vessel permeability. J Vis Exp. 2013;73:e50062. 10.3791/50062 PMC3639515 23524912

[20] Matute-Bello G, Downey G, Moore BB, Groshong SD, Matthay MA, Slutsky AS, Kuebler WM, Acute Lung Injury in Animals Study G (2011). An official American Thoracic Society workshop report: features and measurements of experimental acute lung injury in animals. Am J Respir Cell Mol Biol.

[21] Helwa I, Cai J, Drewry MD, Zimmerman A, Dinkins MB, Khaled ML, Seremwe M, Dismuke WM, Bieberich E, Stamer WD, Hamrick MW, Liu YA (2017). Comparative study of serum exosome isolation using differential ultracentrifugation and three commercial reagents. PLoS One.

[22] Wu XJ, Zhao ZF, Kang XJ, Wang HJ, Zhao J, Pu XM (2016). MicroRNA-126-3p suppresses cell proliferation by targeting PIK3R2 in Kaposi’s sarcoma cells. Oncotarget.

[23] Wu X, Su D (2018). Enterotoxigenic Escherichia coli infection induces tight junction proteins expression in mice. Iran J Vet Res.

[24] Harhaj NS, Antonetti DA (2004). Regulation of tight junctions and loss of barrier function in pathophysiology. Int J Biochem Cell Biol.

[25] Ranghino A, Cantaluppi V, Grange C, Vitillo L, Fop F, Biancone L, Deregibus MC, Tetta C, Segoloni GP, Camussi G (2012). Endothelial progenitor cell-derived microvesicles improve neovascularization in a murine model of hindlimb ischemia. Int J Immunopathol Pharmacol.

[26] Cantaluppi V, Gatti S, Medica D, Figliolini F, Bruno S, Deregibus MC, Sordi A, Biancone L, Tetta C, Camussi G (2012). Microvesicles derived from endothelial progenitor cells protect the kidney from ischemia-reperfusion injury by microRNA-dependent reprogramming of resident renal cells. Kidney Int.

[27] Lu Y, Zhang H, Teng F, Xia WJ, Sun GX, Wen AQ (2018). Early goal-directed therapy in severe sepsis and septic shock: a meta-analysis and trial sequential analysis of randomized controlled trials. J Intensive Care Med.

[28] Chu M, Qin S, Wu R, Zhou X, Tang X, Zhang S, Zhao Q, Wang H, Liu Y, Han X, Xiao J, Li X, Zhang C (2016). Role of miR-126a-3p in endothelial injury in endotoxic mice. Crit Care Med.

[29] Lu J, Wang X, Chen Q, Chen M, Cheng L, Dai L, Jiang H, Sun Z (2016). The effect of early goal-directed therapy on mortality in patients with severe sepsis and septic shock: a meta-analysis. J Surg Res.

[30] Zhuang Y, Peng H, Mastej V, Chen W (2016). MicroRNA regulation of endothelial junction proteins and clinical consequence. Mediat Inflamm.

[31] Xi T, Jin F, Zhu Y, Wang J, Tang L, Wang Y, Liebeskind DS, He Z (2017). MicroRNA-126-3p attenuates blood-brain barrier disruption, cerebral edema and neuronal injury following intracerebral hemorrhage by regulating PIK3R2 and Akt. Biochem Biophys Res Commun.

[32] Zhou Y, Geng X, Chen Y, Shi H, Yang Y, Zhu C, Yu G, Tang Z (2018). Essential roles of Akt/Snail pathway in microcystin-LR-induced tight junction toxicity in Sertoli cell. Food Chem Toxicol.

[33] Huang W, Zhao H, Dong H, Wu Y, Yao L, Zou F, Cai S (2016). High-mobility group box 1 impairs airway epithelial barrier function through the activation of the RAGE/ERK pathway. Int J Mol Med.

